# Serum Vitamin E Levels of Adults with Nonalcoholic Fatty Liver Disease: An Inverse Relationship with All-Cause Mortality in Non-Diabetic but Not in Pre-Diabetic or Diabetic Subjects

**DOI:** 10.3390/jcm8071057

**Published:** 2019-07-19

**Authors:** Peiling Tsou, Chang-Jiun Wu

**Affiliations:** Department of Genomic Medicine, University of Texas, MD Anderson Cancer Center. 1901 East Road, 3SCR5.4101, Houston, TX 77054, USA

**Keywords:** nonalcoholic fatty liver disease (NAFLD), nonalcoholic steatohepatitis (NASH), diabetes, vitamin E, diabetes all-cause mortality, NHANES

## Abstract

Nonalcoholic fatty liver disease (NAFLD) is a growing health threat worldwide. Vitamin E supplementation is recommended for nonalcoholic steatohepatitis (NASH) patients, but only for non-diabetic subjects. We aimed to investigate whether serum vitamin E levels differently impact long-term prognosis in diabetic versus non-diabetic NAFLD individuals. A total of 2404 ultrasonographically defined NAFLD individuals from National Health and Nutrition Examination Survey (NHANES) III were stratified by their glycemic statuses into diabetic (*N* = 662), pre-diabetic (*N* = 836) and non-diabetic (*N* = 906), and the relationship between serum vitamin E levels and all-cause mortality was analyzed. The serum vitamin E concentrations were 31.1 ± 14.1, 26.7 ± 9.6, and 24.7 ± 9.8 µmol/L and vitamin E: total cholesterol ratios were 5.16 ± 1.70, 4.81 ± 1.46, and 4.80 ± 1.34 µmol/mmol in in diabetic, pre-diabetic, and non-diabetic groups, respectively. Of 2404 NAFLD subjects, 2403 have mortality information and 152 non-diabetic, 244 pre-diabetic, and 342 diabetic participants died over a median follow-up period of 18.8 years. Both serum vitamin E levels and vitamin E: total cholesterol ratios were negatively associated with all-cause mortality after adjusting for possible confounders in non-diabetic subjects (HR = 0.483, and 0.451, respectively, *p* < 0.005), but not in either diabetic or pre-diabetic subjects. In NAFLD individuals, both serum vitamin E and lipid-corrected vitamin E were (1) higher in the diabetic group; and (2) negatively associated with all-cause mortality only in the non-diabetic group. Further investigations are warranted to elucidate the underlying mechanism of this inverse association of serum vitamin E concentration with all-cause mortality in non-diabetic but not pre-diabetic or diabetic subjects.

## 1. Introduction

Nonalcoholic fatty liver disease (NAFLD) is the most prevalent form of liver disease in the United States, affecting an estimated 30% of the population. Globally, the prevalence is approximately 25% [1,2]. NAFLD is characterized by accumulation of fat in hepatocytes without specific etiology of secondary hepatic steatosis, such as excessive alcohol consumption, viral hepatitis, or a hereditary disorder [3]. NAFLD encompasses a broad pathological spectrum of phenotypes ranging from hepatic steatosis with no evidence of hepatocellular injury, non-alcoholic fatty liver (NAFL), to nonalcoholic steatohepatitis (NASH)—the progressive form of fatty liver disease associated with inflammation and cellular injury, which can lead to NASH-related cirrhosis and hepatocellular carcinoma [4,5]. Importantly, the number of patients with NASH on the transplantation waiting list increased by 170% from 2004 to 2013, the largest absolute and relative increase compared with other etiologies [6].

To date, no definite pharmacological treatment has been approved for NAFLD. Clinical evidence strongly supports the role of lifestyle modification as a primary therapy for the management of NAFLD and NASH [7]. Moreover, modification of cardiovascular risk factors should be evaluated. For instance, cardiovascular outcome trials with pioglitazone have demonstrated that this insulin sensitizing thiazolidinedione reduces cardiovascular events in high risk type 2 diabetic patients [8]. Also, lipid-lowering agents should also be considered for patients with dyslipidemia [9]. In 2018, a few phase 3 clinical trials for the treatment of NASH have been initiated. Additionally, several phase 2a and 2b clinical trials targeting different pathogenic pathways in NASH are in the pipeline of emerging therapies [10,11].

Although the pathogenesis of NAFLD and its progression to fibrosis needs to be fully clarified, oxidative stress and inflammation are believed to play critical roles in the transition from steatosis to NASH [5,12,13]. Oxidative stress results from an imbalance between production of reactive oxygen species (ROS) and antioxidant defenses. Several efforts exploring anti-oxidative, anti-inflammatory nutraceuticals have been proposed so far, including vitamin C [14], vitamin D [15], and polyunsaturated fatty acids (PUFAs) [16]. However, the only proven effective so far is vitamin E administration [17,18]. Vitamin E is a lipid-soluble, chain-breaking antioxidant that prevents the propagation of free radicals [19,20]. Moreover, vitamin E interacts with other cellular components and may help promote the antioxidative environment. In addition to its anti-oxidative role, vitamin E may help delay hepatic fibrosis and inhibit cirrhosis by modulating inflammatory response and cellular proliferation [21,22]. It has been demonstrated that vitamin E can regulate the expression of specific genes not only coupled to oxidative stress but also involved in inflammatory pathways [23].

Since oxidative stress and inflammation are considered as key mechanisms of hepatocellular damage that contributes disease progression in NAFLD individuals, vitamin E has been extensively investigated as a treatment for NASH. Two well-known randomized clinical trials are the ‘Pioglitazone, Vitamin E, or Placebo for Nonalcoholic Steatohepatitis’ (PIVENS) trial by Sanyal et al. [18], and the ‘Treatment of NAFLD in Children’ (TONIC) pediatric trial by Lavine et al. [17]. The PIVENS trial demonstrated improvement in liver histology in 43% compared to a placebo response of 19% (*P* = 0.001) and similarly the TONIC trial showed resolution of NASH in 58% compared to 28% in placebo (*P* = 0.006). Of note, both the PIVENS and TONIC trials were conducted in patients without diabetes or cirrhosis so the benefits of vitamin E in patients with these common co-morbidities remain unknown. Accordingly, the American Association for the Study of Liver Diseases (AASLD) (Alexandria, VA, USA) guidelines [24] lists vitamin E to be considered only for biopsy-proven non-diabetic patients. Importantly, long-term safety remains a concern for NASH patients under vitamin E therapy since there have been several studies suggesting that high-dosage (≥400 IU/day) vitamin E supplements may increase all-cause mortality [25]. Thus, it is necessary to assess whether vitamin E might impact differently in diabetic versus non-diabetic NAFLD individuals on their prognosis In addition, a link between cardiovascular disease and prediabetes has emerged over the past few years [26]. Likewise, not only type 2 diabetes but also prediabetes is associated with portal inflammation and fibrosis in patients with non-alcoholic fatty liver disease [27]. It has been shown that subjects with HbA1c-defined prediabetes and type 2 diabetes, respectively, are characterized by abnormalities in lipid profile and liver steatosis [28]. Therefore, we also included the group of pre-diabetic subjects into our investigation.

While vitamin E supplements have been investigated in many studies, limited data are available on the relation between serum vitamin E and the long-term prognosis of NAFLD. Since direct measurement of vitamin E level from liver biopsy tissue is too invasive, measurement of blood as an aggregate of both dietary and biological processed of vitamin E may be a more appropriate surrogate for assessing vitamin E effects than dietary intake or supplement dosage assessment.

The primary goal of this study was to examine, in NAFLD subjects with non-diabetes, pre-diabetes, and diabetes, (1) whether serum vitamin levels differ, and (2) whether serum vitamin E associates differently with all-cause mortality, among distinct diabetic status.

## 2. Materials and Methods

### 2.1. Study Population

National Health and Nutrition Examination Survey (NHANES) is a cross-sectional observational study conducted by the National Center for Health Statistics of the Centers for Disease Control and Prevention that uses a stratified, multistage probability design to obtain a nationally representative sample of the U.S. population.

#### 2.1.1. Definition of NAFLD Cases

In the NHANES III cohort [29], 16,115 adults aged 20–74 years old completed hepatic ultrasound examinations. The ultrasound examinations were performed using a Toshiba (Tustin, CA) SSA-90A machine using 3.75 and 5.0 MHz transducer. Briefly, the following information was recorded on a standard paper collection form: (1) the presence of liver-to-kidney contrast (2) the degree of the brightness of the liver parenchyma, (3) the presence of deep beam attenuation, (4) the presence of echogenic walls in the small intrahepatic vessels, and (5) the definition of the gallbladder walls. Finally, an overall primary finding was given based on the presence or absence of each of the five parameters. The liver was then graded as normal, mild, moderate, or severe hepatic steatosis. We only selected images that were categorized as “confident” or “absolute” (*N* = 12,915) and did not include images labeled “ungradable” or “doubtful”. Among these ultrasound scans, we first chose cases with moderate to severe hepatic steatosis (*N* = 2722) and then excluded subjects with alcohol consumption >20 g/day in women and >30 g/day in men [30]. Furthermore, cases with viral hepatitis B and C, those tested positive for hepatitis B surface antigen or positive for anti-HCV antibody were excluded. The remaining 2404 subjects constituted the NAFLD cohort of the current study.

#### 2.1.2. Definition of Diabetic Statuses

Individuals with fasting plasma glucose (FBG) ≥126 mg/dL or the 2nd hour (2h)-oral glucose tolerance test (OGTT) plasma glucose ≥ 200 mg/dL, glycosylated hemoglobin (HbA1c) ≥ 6.5%, use of oral hypoglycemic medications or insulin, and previous history of diabetes were considered to have diabetes. Pre-diabetes was defined as any of the following conditions: 100 ≤ FBG <126 mg/dL, 140 mg/dL ≤ 2h-OGTT plasma glucose <200 mg/dL, or 5.7% ≤ HbA1c < 6.5%. Normal glycemia was defined as FBG < 100 mg/dL, 2h-OGTT plasma glucose < 140 mg/dL, and HbA1c < 5.7%.

### 2.2. Examinations and Laboratory Measurements

#### 2.2.1. Serum Vitamin E Measurement and Correction by Cholesterol Levels

Serum vitamin E (alpha-tocopherol) levels were measured by isocratic high-performance liquid chromatography with detection at three different wavelengths [31]. Extreme values 0.1% from the top were excluded as outliers. Lipid-corrected vitamin E (i.e., vitamin E: lipid ratio) was calculated by taking serum vitamin E concentration in μmol/L and divided by plasma total cholesterol concentration in mmol/L [32].

#### 2.2.2. Other Covariates

Body mass index (BMI) was calculated using the formula: BMI = kg/m^2^ where kg is body weight in kilograms and m^2^ is body height in meters squared. Hypertension was determined if the mean systolic blood pressure >130 mmHg or the mean diastolic >85 mmHg. Serum cotinine is a major metabolite of nicotine and a biomarker of tobacco smoke exposure. The enzyme immunoassay (EIA) method was used as a screening method for and liquid chromatography-mass spectrometry was applied for confirmational measurements. Individuals with serum cotinine levels > 10 ng/mL were coded as being current smokers.

Fibrosis-4 (FIB4) scores were calculated for all NAFLD subjects using the previously published formula [33].

FIB4 score = Age (year) × AST (IU/L)/(platelet count (10^9^ /L) × square-root of ALT (IU/L)) (1)

### 2.3. Mortality Status and Follow-Up Duration

Mortality status for adult NHANES III participants was reported as of 31 December 2011 by NCHS through the US National Death Index (NDI), which is a computerized database of all certified deaths in the USA since 1979. The NHANES III-Linked Mortality File was downloaded [4] and all-cause mortality information was used in the analysis. Follow-up length was calculated as a period between examination for NHANES III and death or the end of follow-up, whichever came earlier.

### 2.4. Statistical Methods

Descriptive statistics of subjects’ characteristics were reported, including means and standard deviations for continuous variables as well as percentages for categorical variables. Comparison of variables among the three glycemic groups was assessed using Kruskal-Wallis test [34] for continuous variables and Fisher’s exact test [35] for categorical variables. *p*-Values < 0.05 were considered statistically significant.

To examine if serum vitamin E is associated with all-cause mortality among different risk groups, we used various multivariate Cox proportional hazards regression models to estimate the hazard ratios of serum vitamin E (logarithmically transformed based on 2) and different combinations of covariates including age, gender, smoking status, HbA1c and FIB4 score. Considering the influence of lipid concentration on functional vitamin E level, we repeated Cox regression analyses using log-2 transformed lipid-corrected vitamin E, defined by vitamin E: cholesterol ratios (μmol: mmol). Forest plots show the hazard ratios and 95% confidence intervals of covariates in each model. Analyses were conducted using the *coxph* function of the *survival* package in R software (R version 3.3.0, Vienna, Austria).

## 3. Results

### 3.1. Subject Characteristics

Table 1 presents the demographic, anthropometric, and biochemical characteristics of the whole study population (all NAFLD, *n* = 2404), as well as three glycemic groups stratified by diabetic status (non-diabetic, *n* = 906; pre-diabetic, *n* = 836; and diabetic, *n* = 662). The mean age of all NAFLD cases was 48.3 years. Individuals with diabetes were older (57.3 ± 12.7) than pre-diabetic (51.0 ± 14.1) and non-diabetic subjects (39.1 ± 13.8). As expected, diabetic subjects not only had worse glycemic status and insulin resistance by definition, but also exhibited a higher percentage of other metabolic derangement, including higher blood pressure, higher BMI and worse lipid profile (Table 1). The percentage of current smoking (defined by serum cotinine level) was, on average, 26.9% in the whole NAFLD cohort, while the diabetic group had a lower rate of 21.9%. With regard to liver biochemistry profile, total bilirubin, aspartate aminotransferase (AST) and alanine aminotransferase (ALT) did not significantly differ among the 3 groups, but alkaline phosphatase (ALP) and gamma glutamyltransferase (r-GT) gradually increased from non-diabetic, pre-diabetic, to overt diabetic status (Table 1).

We then examined whether or not the severity of liver damage, determined by a non-invasive Fibrosis 4 (FIB4) score, was different among the 3 glycemic groups. Using the published criteria of FIB4 score [33], we categorized the extent of hepatic fibrosis into mild: F0–F1 (FIB4 < 1.3), moderate: F2 (1.3 < FIB4 < 2.67), and advanced fibrosis: F3 (FIB4 > 2.67). The majority (90.6%) of non-diabetic subjects were classified as F0–F1, 9.1% were F2, and very few (0.3%) were F3. In contrast, nearly a quarter (25.6%) and a third (34.2%) were already F2 or F3 in the pre-diabetic and diabetic groups, respectively.

### 3.2. Serum Vitamin E Levels

Serum vitamin E levels were 24.7 ± 9.8, 26.7 ± 9.6, 31.1 ± 14.1 µmol/L in the non-diabetic, pre-diabetic and diabetic groups, respectively (*p* < 0.001). Since the diabetic group was older and had higher lipoproteins, and both aging and dyslipidemia were known to increase plasma vitamin E lipoprotein carriers [36,37], we thus also calculated lipid-corrected vitamin E levels, defined by vitamin E:cholesterol ratios (µmol:mmol). As listed in Table 1, the corrected vitamin E level remained significantly higher in the diabetic group (5.16 ± 1.70) than in both the non-diabetic (4.81 ± 1.46), and pre-diabetic (4.80 ±1.34) groups (*p* < 0.001).

### 3.3. The Association of Serum Vitamin E Levels and All-Cause Mortality

**Figure 1:**
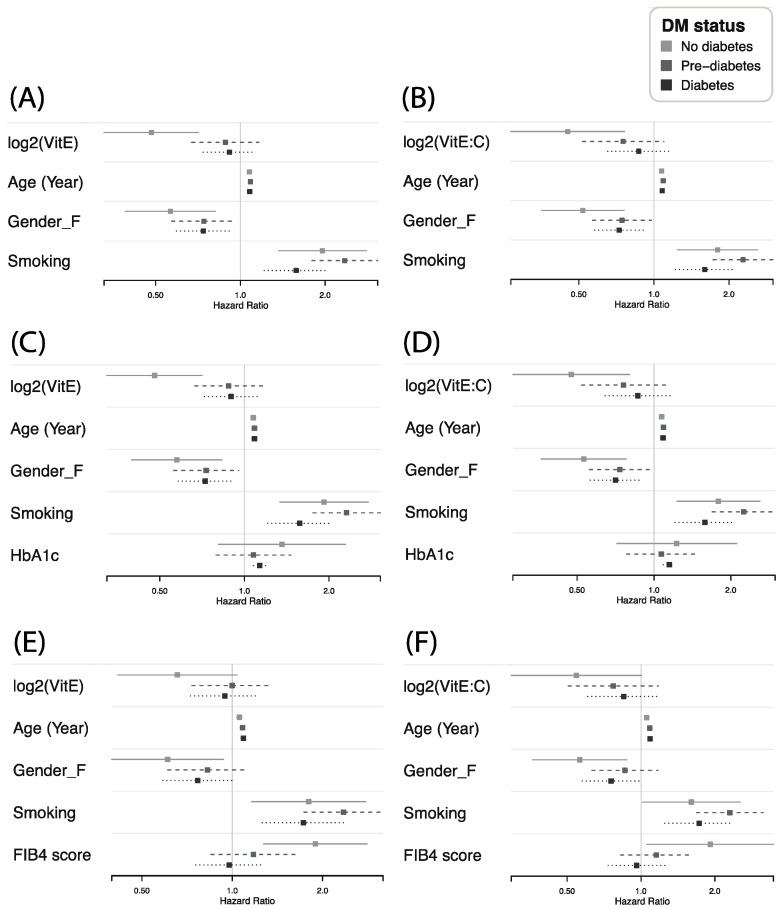
Comparison of hazard ratios in 3 diabetic status groups of serum vitamin E (**A,C,E**) or vitamin E: cholesterol ratio (**B,D,F**) with various models adjusting for potential contributing factors on all-cause mortality. (**A**) Serum vitamin E (**B**) serum vitamin E: cholesterol ratio was inversely correlated with mortality only in non-diabetic group (**C**) Serum vitamin E as well as (**D**) serum vitamin E:cholesterol ratio was inversely correlated with mortality only in non-diabetic group, while HbA1c is positively associated with higher risk only in diabetic group (**E,F**) FIB4 score was positively correlated with mortality only in non-diabetic group. The effect of serum vitamin E (**E**) or lipid-corrected vitamin E level (**F**) on mortality decreases while FIB4 score was added as a covariate in the model (compare **A** vs. **E**, and **B** vs. **F**).

Of the 2404 NAFLD study participants, 2403 have mortality information and 738 died over a median follow-up period of 18.8 years. There were 152, 244, and 342 deaths in the non-, pre-, and diabetic groups, respectively. Table 2 and Table 3 present the survival analysis results on the relationship of all-cause overall mortality and serum vitamin E level (Table 2) or lipid-corrected vitamin E (Table 3) utilizing proportional hazard models to adjust the confounding effects of various covariates. Forest plots in Figure 1 illustrate the comparisons of hazard ratios of potential contributing factors in the 3 diabetic status groups. In all models tested, female gender was consistently anti-correlated, while age and smoking were positively correlated with higher all-cause mortality. Specifically, as shown in Figure 1A, serum vitamin E was inversely correlated with mortality only in the non-diabetic group in the model including age, gender, smoking as covariates (model 1). This holds true in Figure 1B, when corrected vitamin E was tested instead. Consistently, when glycosylated hemoglobin (HbA1c) was added into the model, both serum vitamin E (Figure 1C) and corrected level (Figure 1D) remained significantly anti-correlated with mortality only in non-diabetic group (model 2: *p* < 0.001 in Table 2 and *p* = 0.006 in Table 3). Not surprisingly, HbA1c was associated with shorter survival only in diabetic group (*p* < 0.001, model 2 in Table 2 and Table 3). Furthermore, as shown in Figure 1E,F, when FIB4 score was included as a covariate in the model, it was related with worse prognosis only in non-diabetic group (model 3: *p* = 0.002 in Table 2 and *p* = 0.031 in Table 3). Notably, the significance of serum vitamin E with mortality in non-diabetic individuals decreased (model 1 vs. model 3: HR = 0.48, *p* < 0.001 vs. HR = 0.66, *p* = 0.071 in Table 2; and HR = 0.45, *p* = 0.003 vs. HR = 0.55, *p* = 0.052 in Table 3), suggesting that the effect of vitamin E on mortality might be, at least in part, related to the severity of liver conditions.

## 4. Discussion

The bi-directional associations between NAFLD and type 2 diabetes have been well recognized [29,38,39]. The prevalence of NAFLD in type 2 diabetes is reportedly around one third to two thirds [40,41], and approximately 18 million people in the U.S. have coexisting type 2 diabetes and NAFLD [42]. However, while vitamin E has been shown to improve nonalcoholic steatohepatitis (NASH) in patients without diabetes, information on patients with type 2 diabetes mellitus (T2DM) has just begun to be explored. This current study shows that in NAFLD adults, the serum concentrations of vitamin E, both corrected and non-corrected, were 1) higher in the diabetic group, and 2) negatively associated with all-cause mortality in non-diabetic but not in hyperglycemia or in diabetic individuals. Although the analysis should not directly translate into a specific benefit of vitamin E supplementation for non-diabetic NAFLD, our results, nevertheless, were indeed in line with the 2018 guidance from AASLD that “*until further supporting its effectiveness become available, vitamin E is not recommend to treat NASH in diabetic patients*” [43]. Consistently, a recent randomized controlled trial also showed that vitamin E alone did not significantly change the primary histological outcome in patients with NASH and type 2 diabetes [44].

While vitamin E supplements have been studied in many clinical trials, limited data are available on the association between serum vitamin E concentration and the long-term prognosis of NAFLD. Assessment of dietary intake or oral supplement of with varied dosages and preparations, thought mostly often used, do not reflect the inter-individual differences in absorption, metabolism, tissue distribution, and other genetic variations leading to different levels of circulating vitamin E and may not always guarantee an adequate bioavailability. Nevertheless, direct measurement of vitamin E level from liver biopsy tissue is too invasive. Therefore, measurement of blood as an aggregate of both diet and biologically processed vitamin E, followed by presenting vitamin E as a ratio of circulating lipid concentration [32,37] may be a more appropriate way to determine vitamin E status in our subjects.

It is intriguing that the negative correlation between all-cause mortality and serum vitamin E was only seen in the non-diabetic group. Circulating vitamin E concentrations can sometimes be difficult to interpret [32,37]. For the majority of samples, measurement of serum vitamin E concentration alone is sufficient to establish actual subject vitamin E status [32]. However, in our study cohort, the diabetic group was older and had a worse lipid profile than the non-diabetic group. Aging and dyslipidemia both increase plasma lipoprotein carriers that carry vitamin E, leading to higher circulating vitamin E levels; yet abnormal lipoprotein metabolism does not necessarily increase vitamin E delivery to tissues. It has been documented that α-tocopherol bioavailability is lower in adults with metabolic syndrome [45] and metabolic syndrome increases dietary α-tocopherol requirements as assessed using urinary and plasma vitamin E catabolites [46]. In addition, circulating vitamin E (α- and γ-tocopherol/cholesterol ratio) levels are shown to be positively associated with metabolic syndrome and visceral fat volumes [47]. It is possible that, similar to the scenario with metabolic syndrome, higher circulating lipid concentrations and slower α-tocopherol turnover of diabetic subjects artificially elevated plasma α-tocopherol concentrations, which led to the erroneous impression that these subjects had higher vitamin E levels. Also, the inner environment in diabetes is likely more inflammatory, and that the compensatory higher serum vitamin E level simply reflects a higher demand of antioxidants. In addition, yet under extremely inflammatory situation, vitamin E might not be functioning as well as in less inflammatory milieu.

Moreover, since the FIB4 scores were lower in the non-diabetic group, it is also possible that the favorable effects of vitamin E were only applicable when the extent of liver damage is mild. When the insult becomes too severe, it may overwhelm the anti-oxidant and anti-inflammatory benefits of vitamin E. Indeed, in non-diabetic group, the correlation of higher serum vitamin E with better survival slightly decreased when FIB4 score were taken into consideration, suggesting that the beneficial effect of vitamin E on mortality might be, at least in part, through the ameliorating the extent of NAFLD.

The present study has a few limitations. It is important to point out that since long-term follow-up liver biochemistry or biopsy data was not available, whether higher serum vitamin E levels prevent the progression of NAFLD, biochemically or histologically, were not known. Thus, the benefit on mortality could not be claimed as liver-specific. Since a great portion of our non-diabetic NAFLD cohort also exhibits various metabolic derangements, it is possible that anti-oxidative and anti-inflammatory benefits operate more broadly, and the reduced mortality was attributed to, for example, decreased cardiovascular events or even cancer related death [48]. Further researches with specifically defined primary end-points are required to investigate factors that may modulate effects of vitamin E on all-cause mortality. Second, although we adjusted for factors that might have an impact on all-cause mortality, we cannot exclude the role of residual or unmeasured confounding in the findings. Last but not least, although NHANES uses a stratified, multistage probability design to obtain a nationally representative sample of the U.S. population, the subsample was chosen by data availability of ultrasound scans and serum vitamin E levels from the NHANES cohort. Therefore, it takes extra cautions to generalize the results.

## 5. Conclusions

To summarize, in NAFLD individuals both serum vitamin E and lipid-corrected vitamin E were (1) higher in the diabetic group, and (2) negatively associated with all-cause mortality only in non-diabetic, and not in pre-diabetic or diabetic individuals. The mechanism underlying the inverse relationship remains to be elucidated and further research may provide important insights for patient-tailored therapy targeting this growing health burden worldwide.

## Figures and Tables

**Table 1:** Characteristics of all NAFLD subjects and of three subgroups stratified by diabetic status.

	All NAFLD		Diabetic Status						* *p-*Value
	*n* = 2404		Non-Diabetic (*n* = 906)		Pre-Diabetic (*n* = 836)		Diabetic (*n* = 662)		
	Mean ± SD	*n*	Mean ± SD	*n*	Mean ± SD	*n*	Mean ± SD	*n*	
Age (year)	48.3 ± 15.6	2404	39.1 ± 13.8	906	51.0 ± 14.1	836	57.3 ± 12.7	662	<0.001
Gender (M, %)	49.2	2404	46.5	906	55.3	836	45.2	662	§ <0.001
Smoking (%)	26.9	2287	28.8	906	28.9	836	21.9	662	§ 0.003
Weight Circumference (cm)	101 ± 15.5	2311	95.4 ± 16.5	878	103 ± 13.9	800	106 ± 13.5	633	<0.001
Body Mass Index (kg/m^2^)	30.1 ± 6.6	2394	28.7 ± 6.8	903	30.8 ± 6.2	832	31.3 ± 6.3	662	<0.001
Hypertension (%)	45.2	2401	28.1	903	51.9	832	60.1	662	§ <0.001
Systolic Blood Pressure (mmHg)	128 ± 19	2401	121 ± 16	903	130 ± 18	832	136 ± 20	662	<0.001
Diastolic Blood Pressure (mmHg)	77 ± 11	2401	76 ± 11	903	79 ± 11	832	77 ± 11	662	<0.001
Plasma Triglyceride (mg/dL)	179 ± 108	2195	153 ± 101	796	177 ± 100	797	216 ± 116	602	<0.001
Plasma HDL (mg/dL)	45.6 ± 12.6	2195	47.1 ± 13.2	796	45.2 ± 12.3	797	44.2 ± 11.9	602	<0.001
Plasma Total cholesterol (mg/dL)	209 ± 41.3	2195	196 ± 41	796	212 ± 39	797	221 ± 40.7	602	<0.001
Fasting Plasma Glucose (mg /dL)	114 ± 54.8	2328	90.1 ± 6.5	839	100 ± 9.9	833	164 ± 83.9	656	<0.001
Glycated hemoglobin (%), HbA1c	5.93 ± 1.54	2327	5.10 ± 0.37	838	5.55 ± 0.45	834	7.47± 2.13	655	<0.001
HOMA-IR	0.576 ± 1.12	2304	0.294 ± 0.264	831	0.436 ± 0.389	828	1.12 ± 1.95	645	<0.001
Serum total protein(g/dL)	7.43 ± 0.44	1915	7.42 ± 0.44	686	7.42 ± 0.43	716	7.47 ± 0.44	513	0.200
Serum Albumin (g/dL)	4.15 ± 0.36	1915	4.18 ± 0.36	686	4.16 ± 0.36	716	4.09 ± 0.34	513	<0.001
Serum Globulin (g/dL)	3.33 ± 0.43	1503	3.27 ± 0.41	535	3.33 ± 0.43	554	3.41 ± 0.45	396	<0.001
Aspartate aminotransferase (U/L)	24.4 ± 14.2	1915	23.7 ± 11.8	686	24.8 ± 12.6	716	24.9 ± 18.5	513	0.518
Alanine aminotransferase (U/L)	23.9 ± 19.7	1915	23.4 ± 19.6	686	24.6 ± 19.3	716	23.7 ± 20.1	513	0.918
Alkaline phosphatase (U/L)	90.6 ± 28.1	1915	85.2 ± 23.5	686	90.9 ± 28.6	716	97.6 ± 31.3	513	<0.001
Gamma glutamyl transferase (U/L)	40.1 ± 46.8	1542	31.9 ± 41.7	564	41.7 ± 35.5	569	49.0 ± 62.7	409	<0.001
Serum Total Bilirubin (mg/dL)	0.585 ± 0.257	1915	0.588 ± 0.267	686	0.578 ± 0.249	716	0.591 ± 0.257	513	0.230
Platelet Counts (^10^9^/L)	279 ± 69.1	2175	282 ± 67.6	787	278 ± 68.3	782	276 ± 71.8	606	0.340
FIB4 score	0.977 ± 0.542	1798	0.775 ± 0.461	647	1.038 ± 0.515	675	1.165 ± 0.591	476	<0.001
Serum Vitamin E level (µmol/L)	27.4 ± 12.6	2300	24.7 ± 9.84	825	26.7 ± 9.59	827	31.1 ± 14.1	645	<0.001
vitamin E:cholesterol ratio	4.90 ± 1.50	2185	4.81 ± 1.46	791	4.80 ± 1.34	795	5.16 ± 1.70	599	<0.001

* Kruskal-Wallis test unless otherwise specified. **§** Fisher’s exact test. Abbreviations: NAFLD: Non-Alcoholic Fatty Liver Disease; HDL: High Density Lipoprotein; HOMA-IR: HOmeostatic Model Assessment-Insulin Resistance. FIB4 score = age (year) × AST (IU/L)/(platelet count × square root (ALT (IU/L)).

**Table 2:** Serum vitamin E-based Cox regression models on overall mortality in three NAFLD groups stratified by diabetic status.

		Non-Diabetic					Pre-Diabetic					Diabetic				
		coef	HR	Lower CI	Upper CI	*p*	coef	HR	Lower CI	Upper CI	*p*	coef	HR	Lower CI	Upper CI	*p*
**Model 1**	log2(vitE)	−0.729	0.483	0.328	0.710	<0.001	−0.122	0.885	0.672	1.165	0.384	−0.090	0.914	0.738	1.132	0.409
	Age_Yr	0.074	1.077	1.064	1.090	<0.001	0.083	1.086	1.072	1.100	<0.001	0.077	1.080	1.067	1.093	<0.001
	Gender_F	−0.573	0.564	0.390	0.814	0.002	−0.299	0.742	0.570	0.966	0.026	−0.303	0.739	0.593	0.920	0.007
	Smoking_Y	0.672	1.958	1.368	2.803	<0.001	0.854	2.349	1.796	3.074	<0.001	0.457	1.579	1.215	2.053	0.001
		# *n* Cases = 817, *n* Events = 131; log rank *P* < 0.0001					# *n* Cases = 823, *n* Events = 235; log rank *P* < 0.0001					# *n* Cases = 634, *n* Events = 325; log rank *P* < 0.0001				
**Model 2**	log2(vitE)	−0.736	0.479	0.324	0.708	<0.001	−0.129	0.879	0.666	1.158	0.359	−0.110	0.896	0.721	1.113	0.319
	Age_Yr	0.073	1.075	1.062	1.089	<0.001	0.082	1.086	1.072	1.100	<0.001	0.083	1.086	1.073	1.100	<0.001
	Gender_F	−0.553	0.575	0.397	0.832	0.003	−0.313	0.731	0.560	0.953	0.021	−0.322	0.725	0.581	0.904	0.004
	Smoking_Y	0.653	1.920	1.337	2.759	<0.001	0.838	2.311	1.754	3.045	<0.001	0.453	1.573	1.210	2.047	0.001
	HbA1c	0.308	1.360	0.808	2.289	0.247	0.074	1.077	0.793	1.463	0.634	0.126	1.134	1.077	1.195	<0.001
		# *n* Cases = 813, *n* Events = 130; log rank *P* < 0.0001					# *n* Cases = 821, *n* Events = 233; log rank *P* < 0.0001					# *n* Cases = 632, *n* Events = 323; log rank *P* < 0.0001				
**Model 3**	log2(vitE)	−0.422	0.656	0.415	1.036	0.071	−0.001	0.999	0.734	1.361	0.996	−0.056	0.945	0.726	1.231	0.676
	Age_Yr	0.055	1.056	1.036	1.076	<0.001	0.079	1.082	1.064	1.102	<0.001	0.085	1.089	1.071	1.107	<0.001
	Gender_F	−0.495	0.609	0.397	0.935	0.023	−0.190	0.827	0.610	1.121	0.221	−0.266	0.767	0.587	1.002	0.051
	Smoking_Y	0.585	1.796	1.158	2.784	0.009	0.853	2.347	1.733	3.179	<0.001	0.546	1.726	1.259	2.366	0.001
	FIB4 score	0.637	1.891	1.270	2.814	0.002	0.163	1.177	0.849	1.630	0.328	−0.024	0.977	0.756	1.263	0.858
		# *n* Cases = 639, *n* Events = 99; log rank *P* < 0.0001					# *n* Cases = 672, *n* Events = 183; log rank *P* < 0.0001					# *n* Cases = 464, *n* Events = 229; log rank *P* < 0.0001				

The effects of various combinations of potential confounding factors were adjusted with multivariate models as follows: Model 1: survival (month) ~ log2 (vitamin E) + Age (Year) + Gender female + Smoking; Model 2: survival (month) ~ log2 (vitamin E) + Age (Year) + Gender female + Smoking + HbA1c; Model 3: survival (month) ~ log2 (vitamin E) + Age (Year) + Gender female + Smoking + FIB4 scores. Abbreviations: # *n* Cases: number of cases; *n* Events: number of all-cause death events; coef: beta coefficient; CI: 95% confidence interval; HR: Hazard Ratio; log2(vitE): log2(vitamin E levels); Gender_F: female gender; Age_Yr: Age in years; Smoking_Y: current smoking _yes.

**Table 3:** Lipid-corrected vitamin E-based Cox regression models on overall mortality in three NAFLD groups stratified by diabetic status.

		Non-Diabetic					Pre-Diabetic					Diabetic				
		coef	HR	Lower CI	Upper CI	*p*	coef	HR	Lower CI	Upper CI	*p*	coef	HR	Lower CI	Upper CI	*p*
**Model 1**	log2(vitE:C)	−0.796	0.451	0.267	0.763	0.003	−0.283	0.753	0.518	1.096	0.139	−0.140	0.869	0.653	1.156	0.336
	Age_Yr	0.071	1.074	1.061	1.088	<0.001	0.086	1.089	1.075	1.104	<0.001	0.077	1.080	1.066	1.094	<0.001
	Gender_F	−0.656	0.519	0.355	0.759	0.001	−0.294	0.745	0.568	0.977	0.034	−0.320	0.726	0.579	0.911	0.006
	Smoking_Y	0.588	1.799	1.241	2.609	0.002	0.823	2.276	1.726	3.002	<0.001	0.468	1.597	1.219	2.091	0.001
		# *n* Cases = 783, *n* Events = 123; log rank *P* < 0.0001					# *n* Cases = 791, *n* Events = 226; log rank *P* < 0.0001					# *n* Cases = 589, *n* Events = 301; log rank *P* < 0.0001				
**Model 2**	log2(vitE:C)	−0.747	0.474	0.280	0.802	0.006	−0.274	0.760	0.521	1.108	0.154	−0.147	0.864	0.644	1.159	0.329
	Age_Yr	0.070	1.072	1.059	1.086	<0.001	0.085	1.089	1.075	1.103	<0.001	0.083	1.086	1.072	1.101	<0.001
	Gender_F	−0.633	0.531	0.362	0.779	0.001	−0.308	0.735	0.559	0.966	0.027	−0.346	0.707	0.563	0.889	0.003
	Smoking_Y	0.581	1.788	1.230	2.600	0.002	0.809	2.246	1.692	2.982	<0.001	0.460	1.584	1.209	2.076	0.001
	HbA1c	0.206	1.228	0.715	2.110	0.456	0.066	1.068	0.782	1.459	0.680	0.138	1.148	1.088	1.212	<0.001
		# *n* Cases = 779, *n* Events = 122; log rank *P* < 0.0001					# *n* Cases = 789, *n* Events = 224; log rank *P* < 0.0001					# *n* Cases = 587, *n* Events = 299; log rank *P* < 0.0001				
**Model 3**	log2(vitE:C)	−0.607	0.545	0.296	1.005	0.052	−0.261	0.771	0.505	1.176	0.227	−0.163	0.850	0.607	1.190	0.344
	Age_Yr	0.054	1.055	1.033	1.078	<0.001	0.084	1.087	1.068	1.107	<0.001	0.085	1.089	1.070	1.108	<0.001
	Gender_F	−0.575	0.563	0.362	0.875	0.011	−0.150	0.860	0.631	1.174	0.343	−0.282	0.755	0.576	0.988	0.041
	Smoking_Y	0.473	1.604	1.014	2.539	0.044	0.834	2.302	1.684	3.146	<0.001	0.546	1.726	1.251	2.379	0.001
	FIB4 score	0.651	1.917	1.060	3.468	0.031	0.143	1.154	0.826	1.613	0.401	−0.041	0.960	0.735	1.253	0.762
		# *n* Cases = 614, *n* Events = 92; log rank *P* < 0.0001					# *n* Cases = 650, *n* Events = 176; log rank *P* < 0.0001					# *n* Cases = 436, *n* Events = 218; log rank *P* < 0.0001				

The effects of various combinations of potential confounding factors were adjusted with multivariate models as follows: Model 1: survival (month) ~ log2(vitamin E:Cholesterol ratios) + Age (Year) + Gender female + Smoking; Model 2: survival (month) ~ log2(vitamin E:Cholesterol ratios) + Age (Year) + Gender female + Smoking + HbA1c; Model 3: survival (month) ~ log2(vitamin E:Cholesterol ratios) + Age (Year) + Gender female + Smoking + FIB4 scores. Abbreviations: # *n* Cases: number of cases; *n* Events: number of all-cause death events; coef: beta coefficient; CI: 95% confidence interval; HR: Hazard Ratio; log2(vitE:C): log2(vitamin E/Cholesterol); gender_F: female gender; Age_Yr: Age in years; Smoking_Y: current smoking _yes.
